# Safety and pharmacokinetics of a highly bioavailable resveratrol preparation (JOTROL ^TM^)

**DOI:** 10.1186/s41120-022-00058-1

**Published:** 2022-06-30

**Authors:** Christopher Kemper, Dariush Behnam, Shaun Brothers, Claes Wahlestedt, Claude-Henry Volmar, Daniel Bennett, Marshall Hayward

**Affiliations:** 1 Pharma Navigators LLC, 25 Dix Lane, Lawrenceville, NJ 08648 USA; 2 Aquanova AG, Darmstadt, Germany; 3 Jupiter Neurosciences Inc., 1001 US Highway North Suite 504, Jupiter, FL 33458 USA; 4 Center for Therapeutic Innovation, University of Miami Miller School of Medicine, Miami, FL 33136 USA; 5 Syneos Health, 301 College Road East, Princeton, NJ USA

**Keywords:** Resveratrol, Pharmacokinetics, Bioavailability, JOTROL^TM^, Neuroinflammation

## Abstract

Resveratrol exhibits a wide range of biological properties, including anti-glycation, antioxidant, anti-inflammation, neuroprotective (including against advanced dementia and Alzheimer’s disease), anti-cancer, and anti-aging activity in experimental models (Galiniak et al., Acta Biochim Pol 66:13-21, 2019). Unfortunately, this compound exhibits low bioavailability and solubility (Galiniak et al., Acta Biochim Pol 66:13-21, 2019), requiring large doses that can cause nausea and GI distress. JOTROL^TM^ is a micellar 10% resveratrol solubilization formulation that is thought to increase bioavailability of resveratrol via lymphatic system absorption. Jupiter Neurosciences (formerly Jupiter Orphan Therapeutics; “Jupiter”) is pursuing the use of resveratrol in mucopolysaccharidosis type 1 (MPS 1), Friedreich’s ataxia, and Alzheimer’s disease/mild cognitive impairment. This paper describes a first in human study (FIH) to evaluate the bioavailability of resveratrol after ascending, single oral doses up to 700 mg resveratrol as JOTROL^TM^. After a single 500 mg dose of JOTROL^TM^, a Cmax of 455 ng/mL was observed, vs. 85 ng/mL Cmax after a 1 g encapsulated dose (Turner et al., Neurology 85:1383-91, 2015) and 1942 ng/mL after a 2.5 g micronized dose (Howells et al., Cancer Prev Res (Phila) 4:1419-1425, 2011). In this study, resveratrol exposures (AUCs and Cmax) increased with increasing doses. This increase appears to be higher than dose-proportional for AUC_0-t_ and Cmax. Resveratrol and its three major conjugates accounted for 40 to 55% of the dose in urine, consistent with a high extent of absorption, but < 1% of drug-related material was intact relative to key metabolites in plasma and urine. Studies in Alzheimer’s patients and in MPS 1 are currently in development to test the effect this improved bioavailability has on those patient populations (Clintrials.gov, NCT04668274, 12/16/2020, https://clinicaltrials.gov/ct2/show/NCT04668274).

**Supplementary Information:**

The online version contains supplementary material available at 10.1186/s41120-022-00058-1.

## Introduction

**Fig. 1:**
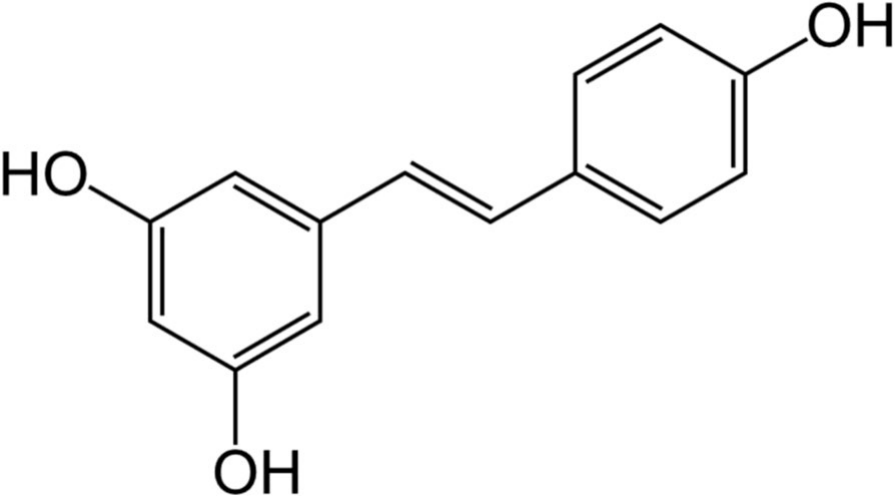
Resveratrol or 3,5,4′-stilbenotriol

Resveratrol or 3,5,4′-stilbenotriol (Fig. 1) is a secondary metabolite present in around 70 plant species, which was isolated for the first time in 1940 from white hellebore (*Veratrum grandiflorum*) root extract (Gambini et al. 2015; Muñoz et al. 2015, Jeandet et al. 2013). It is a phytoalexin, that is, a compound synthesized by plants in response to stress and infection. Structurally, it is a non-flavonoid polyphenol from the stilbene family present in a number of regularly consumed plant species such as berries, peanuts, the epidermis of grapes, and red wine. Its highest concentration is in *Polygonum cuspidatum* roots, a plant mentioned in traditional Chinese and Japanese pharmacopoeias (Aggarwal et al. 2004) and currently used for commercial extraction. Even when biosynthesized in both its cis and trans configurations, a wide consensus considers the trans structure as more biologically active, besides being the most stable isomer (Pantusa et al. 2014). This pharmacological activity of resveratrol has been studied in cardiovascular systems, inflammation, carcinogenesis, aging, diabetes, neurological dysfunction, and lysosomal storage diseases (Galiniak et al. 2019, Aggarwal et al. 2004; Rintz et al. 2020; Yoo and Kim 2015).

One of the biggest challenges in translating the therapeutic effects of resveratrol is the poor bioavailability of the drug when it is administered orally. Resveratrol is eliminated from the body extremely quickly and maintaining a therapeutic level in the bloodstream is difficult (Park and Pezzuto 2015). Most resveratrol is metabolized to glucuronic acid and sulfate conjugations of the phenolic groups in the liver and intestinal epithelial cells and has little pharmacological activity (Wenzel and Somoza 2005; Walle 2011; Wenzel et al. 2005). However, these phase II metabolites may be degraded to the aglycone to form a systemic reservoir of drug.

Various formulations and drug delivery systems have been tested to try to improve the oral bioavailability of resveratrol, but with the exception of multiparticulate formulations, none have shown increased bioavailability (Amri et al. 2012). Results have shown that high doses of resveratrol are needed to affect disease state but can cause serious side effects that limit treatment utilization. JOTROL^TM^ is a micellar 10% resveratrol solubilization formulation that is thought to increase the amount of resveratrol and metabolite concentrations in the blood via the lymphatic system (Behnam and Hayward 2022). A review article on drug delivery to the lymphatic system was published by Ali Khan et al. (2013).

A recent review evaluating resveratrol treatment showed numerous research interests for resveratrol in many indications, including neurological disorders, cardiovascular diseases, and diabetes (Berman et al. 2017). The authors concluded that clinical evidence shows resveratrol favorably influenced disease biomarkers. It is also clear that in many of the clinical trials, the most challenging issue regarding resveratrol development is poor bioavailability.

Previous work in several indications of interest (Friedreich’s ataxia, Alzheimer’s disease) incorporated population pharmacokinetics (PK) and have established a PK target resveratrol Cmax for our JOTROL^TM^ test formulation. In the Yiu et al. (2015) Freidreich’s ataxia study, positive clinical outcomes were associated with a nominal plasma mean Cmax of 261 ng/ml, whereas a nominal mean plasma Cmax level of 128 ng/ml was not associated with any efficacy. In the Alzheimer’s trial, positive biomarker outcomes of amyloid beta and inflammation modulators was associated with a plasma Cmax of 85 ng/ml (Turner et al. 2015; Moussa et al. 2017; Sawda et al. 2017). These data suggest that achieving a target plasma Cmax of 300 ng/ml with a well-tolerated dose of JOTROL^TM^ was a suitable target objective.

The objectives of the PK study described herein were to characterize the PK profile of JOTROL^TM^ (with resveratrol API) following oral administration of single ascending doses (SAD) ranging from 200 mg up to a dose estimated to be 1000 mg in healthy adult subjects. In addition, the effect of food on the PK profile of JOTROL^TM^ was also determined. The Scientific Review Committee (SRC) and independent Data and Safety Monitoring Board (DSMB) reviewed the safety, tolerability, and PK data following completion of each dose level. Dosage levels were based on the plasma levels achieved by the previously reported population PK data and the attainment of target plasma levels at Cmax while not exceeding an AUC level of 2100 ng*h/mL as per discussions with the US Food and Drug Administration. Based on the data from the first 2 study periods, the third period dose was reduced by the SRC to 700 mg and 500 mg was used in the fourth, fed period. The study also evaluated safety and tolerability. Jupiter is pursuing the use of resveratrol as JOTROL^TM^ in MPS 1, Friedreich’s ataxia, MELAS, traumatic brain injury, and Alzheimer’s disease/mild cognitive impairment, among other indications.

## Formulation and drug product development

Details of the formulation are found in the issued patent (15). For production of 1 kg of the solubilization product, 100 g resveratrol; 45 g medium-chain triglycerides; 600 g polysorbate 80; 180 g polysorbate 20, and 75 g mixed tocopherols were used. To protect trans-resveratrol from light degradation, all manufacturing and testing procedures steps were conducted under yellow light.

The resveratrol was (trans-)resveratrol, 99%, CAS number 501-36-0, procured from Evolva, Reinach Switzerland. The CAS number is an international reference standard for chemical substance. Each known chemical substance has a unique CAS number.

Medium chain triglyceride (MCT) oil (70/30) Rofetan GTCC 70/30 made by DHW Deutsche Hydrierwerke Rodleben GmbH, Dessau-RoJlau, Germany, CAS number 73-398-61-5 was used as the medium-chain triglycerides.

Commercial preparations, for example, TEGO SMO 80 V, Evonik or Crillet 4/Tween 80-LQ-(SG), Croda GmbH, Nettetal, Germany, can be used as polysorbate 80 (E433, CAS number 9005-65-6).

Commercial preparations, for example, TEGO SML 20 V, Evonik or Crillet 1/Tween 20-LQ-(SG), Croda GmbH, Nettetal, Germany, can be used as polysorbate 20 (E432, CAS number 9005-64-5). Vitapherole T-70 Non GMO, a 70% mixed tocopherols in plant oil made by Vitae Caps S.A., Spain, or EMix 70 made by Nutrilo GmbH, Cuxhaven, Germany, can be used as mixed tocopherols (E306, CAS numbers 59-02-9, 16698-35-4, 54-28-4, and 119-13-1).

Polysorbate 20, polysorbate 80, mixed tocopherols, and MCT oil were homogenized at a temperature in the range of approximately 18 °C to approximately 22 °C while stirring.

During the course of technology transfer and drug product development, a fill formulation challenge was conducted to assure physical stability. A Temperature Cycling Challenge was conducted by cycling a sample fill solution (20 g) every 24 h from 2 to 80 °C to approximately 40 °C for 7 days. The fill solution was evaluated for physical changes, in which no color change (clear amber), cloudiness, or crystals were observed. A water/plasticizer challenge was conducted by adding different amounts of water or glycerin. The fill solution was evaluated for physical change, in which no color change (clear amber), cloudiness, and no crystals were observed in any of the challenge cases. A One (1) Month Hold Study was conducted by holding the solution in a container at ambient conditions for one (1) month and then inspected for any phase separation or API precipitation. No precipitation or phase separation was observed at the conclusion of this challenge. A fill solution sample was prepared with an API concentration of 12.6% (w/w), equivalent to an additional 2.60% concentration compared to the JOTROL^TM^ formulation. The sample was cooled to room temperature and finally placed at − 19 °C for 48 h. There was no phase separation observed after initial cool down or after the − 19 °C temperature challenge. Finally, Rheological Testing was conducted. The viscosity of the fill solution was evaluated between 10 °C and 60 °C. The fill solution exhibited temperature dependent viscosity between approximately 2000–3000 cP, a unit of measurement for viscosity equivalent to one-hundredth of a poise, at room temperature (22.5 + 2.5 °C). This viscosity this was adequate to allow the fill solution to be encapsulated at room temperature.

Resveratrol was then added to the mixture of polysorbate 20, polysorbate 80, mixed tocopherols, and MCT oil and heated, while stirring to a temperature in the range of approximately 83 °C to approximately 87 °C for homogenization. As soon as the fluid was homogeneous and transparent, it was cooled to a temperature below approximately 30 °C. The solution is then de-aerated by application of vacuum.

The resulting solubilization product is a light brown viscous fluid, which produces a yellowish clear solution when diluted with water at a ratio of 1:50. According to an HPLC analysis, the resveratrol content of the solubilization product is at least 10% by weight, whereby the resveratrol is enclosed in micelles. According to an aerometer measurement, the density of the solubilization product is in the range of 1.05 to 1.15 g/cm^3^ at a temperature of 20 °C. The turbidity of the solubilization product is less than or equal to 50 FNU, solution in water at a ratio of 1:50. The solution has a pH in the range of 6 to 8 according to a potentiometric determination.

Although composition details of the soft gelatin shell are proprietary to the manufacturer (and only referred to in regulatory documents via the closed portion of a Drug Master File), the shell is comprised of standard gelatin (Rousselot) Polysorb (Roquette), anhydrous glycerin (Univar), red and yellow dye (both Sensient), and titanium dioxide as an opacifier (AIC). Red dye and the opacifier were included to protect trans-resveratrol from light.

For the encapsulation process, a Minicaps machine (OET- CMl5001) was used. No heated pump was necessary as the fill material viscosity estimated in the fill material evaluation is sufficient for the fill solution to flow by gravity through the encapsulation equipment train.

The target fill weight per softgel corresponds to the theoretical fill weight of 1.000 g per softgel (fill weight in-process limits of + 3% resulted in fill weight action limit range between 0.910 g and 1.0309 g).

Based on the fill weight of 1.000 g, and the product specific gravity 1.1_,_ the fill volume was estimated at 0.909 mL or 14.755 minims. Based on the fill volume, an Oblong 16 softgel (range from 14.0 to 16.0 minims) was selected. For this ribbon thickness and the selected softgel size and shape, the estimated wet shell weight is 0.692 g. Shell weight in-process limits of + 8% resulted in shell weight action limit range between 0.637 g and 0.747 g.

The encapsulated drug product was placed in shallow trays, and softgels were dried in a controlled condition room (S0140) for up to 9 days. Softgels were sampled and tested for Hardness (by Bareiss Hardness Testing) and Fill Moisture (by Karl Fischer Titration) approximately every 24 h.

Softgels were inspected and transferred to deep trays when average hardness reached 7N (6 days). The remaining softgels were left in shallow trays to continue the hardness and fill moisture testing and then inspected and transferred to deep trays when average hardness reached 9N on day l0.

All softgels were removed from deep trays and washed in a denatured ethanol/phosal 53 MCT wash solution and inspected for leakers (100% inspection).

## Preclinical evaluation

Circulating plasma levels of resveratrol in the formulations as described have shown a surprisingly high level of circulating resveratrol compared to native resveratrol delivered as suspension or delivery of micronized resveratrol API as a suspension as tested in mice and rats. A 5% resveratrol solubilization product-based formula prototype show a higher maximal plasma drug level (“Cmax”) than resveratrol API from the same source and micronized resveratrol from another source. The formulation also showed a higher total absorption amount (the “AUC”). A study in rats at a surprisingly high 10% dose loading showed similarly high plasma levels of resveratrol. These test results are summarized.

Mice were tested for relative plasma bioavailability of resveratrol administered orally from different formulations. At 50 mg/kg resveratrol delivered as the 5% dose loaded solubilization product, the Cmax (average highest maximum concentration in blood plasma) was 17 fold higher than with unformulated API and more than 10 fold higher than micronized Mega Resveratrol. Micronized resveratrol showed slightly higher absorption than standard API. When treated at 25 mg/kg, the solubilization product group showed less than half the resveratrol absorption than the 50 mg/kg dose of the solubilization product but was broadly similar to the 50 mg/kg standard suspension treatments.

The AUC (for 4 h after dosing with 50 mg/kg) was 4 fold higher for the solubilization product than for the Micronized Mega Resveratrol.

In rats, the 10% dose loaded solubilization product dosed at 50 mg/kg showed a Cmax was 7 fold higher than the level observed with micronized resveratrol, and the AUC (for 24 h after dosing) was two and a half fold higher for the solubilization product than for Micronized Mega Resveratrol.

The terminal elimination rate of resveratrol from the solubilization product or from Micronized Mega Resveratrol was the same and consists with literature, meaning that the solubilization product formula does not alter resveratrol metabolism after resveratrol is present in plasma.

In summary, the solubilization product forms of orally administered resveratrol offer superior absorption properties as compared to standard forms. The resveratrol solubilization product formulas clearly outperformed the non-micellar dosing form. Inter-species dose scaling is consistent with expectations, suggesting a dose reduction exploiting the solubilization product is achievable in man.

## Pharmacokinetic study considerations and design

Absorption of micronized resveratrol is good (~ 70%), but the compound is almost totally eliminated by first pass metabolism, primarily to glucuronides and sulfates^22^. To bypass this first pass effect, a micellar formulation was developed to access lymphatic distribution. The structure of the product micelle, with a size of approximately 30 nm, is similar to the structure of the naturally formed physiological mixed micelles containing water-insoluble compounds in its core which is enclosed by ambiphilic molecules (data on file). The physical stability of the JOTROL^TM^ fill material formulation has a minimum shelf life of 12 months (data on file).

### Ethical conduct of the study

All clinical work was conducted in compliance with Good Clinical Practices (GCP) as referenced in the International Council for Harmonization (ICH) guidelines (ICH E6), Good Laboratory Practices (GLP) as referenced in the ICH guidelines, and all applicable regulations, including the Federal Food, Drug and Cosmetic Act, U.S. applicable Code of Federal Regulations (CFR) Title 21, and any IEC requirements relative to clinical studies. The study was also conducted in compliance with the recommendations of the Declaration of Helsinki, with the exception that such registration of phase 1 trials in a publicly accessible database is not mandatory. The protocol, amendments, and informed consent was approved by an Institutional Review Board (IRB, Advarra, and administered via Syneos). An independent Data and Safety Monitoring Board (DSMB) actively participated in the oversight of the study, and the National Institute on Aging participated in study oversight.

This study is intended for submission under FDA regulations. Subjects enrolled in the study will be members of the community at large. Normal healthy male or female volunteers, non-smokers (no use of tobacco products within 3 months prior to screening), ≥ 18 and ≤ 75 years of age, with BMI > 18.5 and < 30.0 kg/m^2^ and body weight ≥ 50.0 kg for males and ≥ 45.0 kg for females. Females of childbearing potential who are sexually active with a male partner followed an acceptable contraceptive method throughout the study. Subjects were excluded from the study if they had any clinically significant abnormalities at physical examination, clinically significant abnormal laboratory test results or positive test for hepatitis B, hepatitis C, or HIV found during medical screening. Positive urine drug screen or urine cotinine test at screening, a history of allergic reactions to resveratrol, polyphenols, other related drugs, or to any excipient in the formulation. Subjects were also excluded if they showed a positive pregnancy test at screening, if they were breast-feeding, subject, or showed clinically significant ECG abnormalities or vital sign abnormalities (systolic blood pressure lower than 90 or over 140 mmHg, diastolic blood pressure lower than 50 or over 90 mmHg, or heart rate less than 50 or over 100 bpm) at screening. Otherwise, the general exclusion criteria will be used (drug or alcohol abuse, use of resveratrol, recent or concomitant enrollment in other clinical studies, use of drugs or supplements known to inhibit hepatic drug metabolism, recent blood donation, or any reason which, in the opinion of the investigator, would prevent the subject from participating in the study).

A total of 24 healthy, adult male or female volunteers were included in study part 1. In part 1, all subjects were sequentially dosed under fasting conditions in an ascending manner across 3 dose levels (200 mg, 500 mg, and 700 mg). A food effect arm was also included as part 2. Key design parameters are summarized in Table 1. While resveratrol is an OTC product, this was the FIH study with the JOTROL^TM^ formulation. As such, the PK of resveratrol in JOTROL^TM^ were unknown and a reasonable estimate of subject number needed was not evaluable. Given that the in life portion of the study was done during the pandemic, total number, gender, ethnicity, etc., of the subjects was not mandated.

**Table 1:** Study drug formulation and test cohorts

	Study period 1 (treatment A)	Study period 2 (treatment B)	Study period 3 (treatment C)	Study period 4 (treatment D)
**Product**	JOTROL^TM^ (resveratrol) gelcaps	JOTROL^TM^ (resveratrol) gelcaps	JOTROL^TM^ (resveratrol) gelcaps	JOTROL^TM^ (resveratrol) gelcaps
**Treatment code**	A	B	C	D
**Strength**	100 mg	100 mg	100 mg	100 mg
**Dosage form**	2 × 100 mg gelcaps	5 × 100 mg gelcaps	7 × 100 mg gelcaps	5 × 100 mg gelcaps
**Dose administered**	200 mg	500 mg	700 mg	500 mg
**Route of administration**	Oral; fasting	Oral; fasting	Oral; fasting	Oral; fed
**Inactive ingredients**	Polysorbate 80, polysorbate 20, mixed tocopherols concentrate, fractionated coconut oil, triglycerides (medium chain)			
**Manufacturer**	Catalent Pharma Solutions, St Petersburg, FL 33716-1016			

A total of 4 study periods were included in this study with washout of at least 14 days between doses. In each study period, subjects were confined to the Syneos Health Clinical Research Facility from day 1 until after the 32-h post-dose blood draw. After the completion of each cohort, an evaluation of the safety data was performed prior to determining whether to proceed with enrollment for the next scheduled dose level, to modify the dose, or to discontinue the study.

### Maximum dose

The FDA preliminary review of the published data on resveratrol found that the nonclinical data could only support daily oral doses of unmodified resveratrol (i.e., non-micronized drug with no absorption enhancers) of up to 3000 mg/day for no more than 13 weeks. Published data from a 26-week study in mice demonstrated renal toxicity at 1000 mg/kg/day, for which the human equivalent dose is approximately 5000 mg. Based on published data in healthy volunteers, AUC exposure at a dose of 3,000 mg/day of unmodified resveratrol was estimated to be 2100 ng•h/mL. Because JOTROL^TM^ formulation has been modified to enhance bioavailability, JOTROL^TM^ doses to be used in this study were to maintain AUC exposures below 2100 ng•h/mL.

### Food and fluid intake

*Study part 1 (periods 1, 2, and 3)*: No food was allowed from at least 10 h before dosing until at least 4 h after dosing

*Study part 2 (period 4)*: After a supervised fast of at least 11 ho, subjects were served a critical, high-fat, high-calorie meal of approximately 800 to 1000 calories (approximately 50% of total caloric content of the meal derived from fat). Drug administration occurred 30 ± 1 m after the meal has been started

Meals were standardized and similar in composition between periods.

Except for fluids provided with the critical breakfast (study part 2 only) and water given with study medication, no fluids were allowed from 1 h before dosing until 1 h post-dose. Water was provided ad libitum at all other times.

### Drug concentration measurements

#### Sample collection and processing

A saline intravenous catheter was used for blood collection to avoid multiple skin punctures. Otherwise, blood samples were collected by direct venipuncture.

The total volume of blood drawn from each subject completing this study did not exceed 400 mL.

#### Blood samples

All blood samples were drawn into blood collection tubes (17 × 3 mL) containing dipotassium ethylenediaminetetraacetic acid (K_2_EDTA) prior to drug administration and 0.133, 0.250, 0.500, 1.00, 1.50, 2.00, 3.00, 4.00, 5.00, 6.00, 8.00, 10.0, 12.0, 16.0, 24.0, and 32.0 h post-dose, during each period. Sample collections done outside the pre-defined time windows (±1 min for samples collected before 8 h post-dose and ± 3 min for subsequent samples) were not considered as protocol deviations since actual post-dose sampling times are used for PK and statistical analyses.

Blood samples were cooled in an ice bath and were centrifuged at 2000 ± 5×g for at least 10 min at approximately 4 °C (no more than 240 min passed between the time of each blood draw and the start of centrifugation). Two aliquots of at least 0.5 mL (when possible) of plasma were dispensed into polypropylene tubes as soon as possible. The aliquots were transferred to a − 80 °C freezer (no more than 60 min passed between the start of centrifugation and aliquot storage), pending analysis/shipment to the analytical facility.

Since resveratrol is sensitive to UV light, blood and plasma collection tubes were protected from light, sample processing was performed under sodium lamp or yellow/gold light, and samples were transferred into amber vials.

At the end of the study, all samples were transferred to the bioanalytical facility (Syneos Health, Princeton, NJ). All transfers between sites were sent in two shipments: one for each set of aliquots. The second shipment was sent only after confirmation of the receipt of the first set of aliquots. Frozen plasma aliquots were sent with sufficient dry ice to maintain the aliquots in a frozen state for at least 72 h. Plasma aliquots were received in good condition and still frozen.

#### Urine samples

Urine samples were collected and pooled according to the following intervals: pre-dose (within 2 h before dosing), 0–4 h, 4–8 h, 8–12 h, 12–24 h, and 24–32 h post-dose. For day 2 (24 h post-dose) urine collection, subjects were asked to void their bladder within 15 min before the end of the collection interval (12–24 h). For other collections, subjects were asked to void their bladder within 10 min before the end of each collection interval. Urine voided at the intersection of two intervals was included in the earlier interval. Any urine voided by subjects but not collected was documented.

The volume of urine collected in each interval was measured (individual urine volumes are on file), and two aliquots of equal volume were dispensed into polypropylene tubes for each interval. Aliquots were stored in a − 80 °C freezer, pending analysis; remaining urine from each subject was discarded.

Urine collection tubes were protected from UV light, sample processing was performed under sodium lamp or yellow/gold light, and samples were transferred into amber vials.

At the end of the study, the first set of frozen urine aliquots from the clinical facility, accompanied by an inventory list and sufficient dry ice to maintain the aliquots in a frozen state for at least 72 h, were sent to the bioanalytical facility (Syneos Health, Princeton, NJ).

Bioanalytical methods for detection and quantitation of resveratrol, resveratrol sulfate, resveratrol 3-glucuronide, and resveratrol 4-glucuronide in human plasma K2EDTA and human urine have been validated in compliance with the FDA 2018 Bioanalytical Method Validation Guidance for Industry.

The method for human plasma has been proven to be precise, accurate, sensitive, and selective over the concentration range studied (5.00 to 5000 ng/mL for resveratrol and resveratrol sulfate and 2.00 to 2000 ng/mL for resveratrol 3-glucuronide and resveratrol 4-glucuronide). Samples are extracted by a protein precipitation extraction procedure, and the compounds are detected and quantified by tandem mass spectrometry in positive ion mode on an MDS Sciex API 6500+ equipped with a Turbo Ionspray® interface. Incurred sample reproducibility was within FDA guidance acceptance criteria for all compounds using this assay.

The method for human urine was proven to be precise, accurate, sensitive, and selective over the concentration range studied (5.00 to 5000 ng/mL for resveratrol, 100 to 100,000 ng/mL for resveratrol sulfate, and 20.0 to 20000 ng/mL for resveratrol 3-glucuronide and resveratrol 4-glucuronide). Samples are extracted by a dilution extraction procedure, and the compounds are detected and quantified by tandem mass spectrometry in positive ion mode on an MDS Sciex API 6500+ (or an MDS Sciex API 4000 for resveratrol sulfate) equipped with a Turbo Ionspray® interface. Incurred sample reproducibility was within FDA guidance acceptance criteria for all compounds using this assay.

All concentration values that were below the lower limit of quantification (BLQ) occurring prior to dosing as well as samples with no reportable value (NRV) occurring prior to dosing were replaced by “0.00”; otherwise, they (BLQ and NRV) were set to missing for tabulation, graphical representation, and calculation purposes. PK analyses were performed using Phoenix WinNonlin® version 8.2, which was validated by Syneos Health. The WinNonlin noncompartmental analysis (NCA) module was used to calculate AUC_0-t_ (last detectable concentration), AUC_0-inf_ (infinity), residual area (%), Cmax, Tmax, T1/2 el (elimination half-life), and Kel (elimination rate constant). Dose proportionality analysis for AUC_0-t_, AUC_0-inf_ and Cmax was performed (using the power model with mixed procedure from SAS®) considering data under fasting conditions (periods 1, 2, and 3). Power model included the PK parameter as the response variable and dose (mg) as the explanatory variable. For this model, the variable dose was treated as a continuous variable. For evaluation of the food-effect, PK data (ln-transformed AUC_0-t_, AUC_0-inf_, Cmax and untransformed Tmax) reported under fed conditions (Period 4) and under fasting conditions (for the same dose level) were compared using analysis of variance (ANOVA) from SAS®. The ratio (fed/fasting) and 90% geometric CI were also calculated for AUC_0-t_, AUC_0-inf_, and Cmax.

The safety data tables and listings, as well as PK tables and listings were created using SAS®, release 9.4 according to FDA guidelines. PK figures were created using WinNonlin® or R version 3.5. The report text was created using Microsoft® Office Word 2016.

## Results

The safety population consisted of 24 subjects who received at least one dose of study medication. Fourteen (14) subjects completed the study, and 10 subjects were discontinued. Of these, 11 subjects completed all 4 treatment periods (treatments A, B, C and D) and received all planned doses, namely, subject nos. 01, 05, 06, 08, 10, 11, 13, 15, 16, 17, and 19.

In part 1 of the study, fifteen (15) subjects received all 3 treatments (treatments A, B, and C), namely, subject nos. 01, 05, 06, 07, 08, 09, 10, 11, 13, 15, 16, 17, 19, 20, and 21.

Of the 24 subjects included in part 1 of the study, a total of 15 subjects (subject nos. 01, 03, 05, 06, 08, 10–13, 15–17, 19, 23, and 24) were enrolled in part 2. Of the 15 subjects enrolled, 14 (93.3%) subjects completed the period 4.

The following subjects received some, but not all, planned treatments:

- Subject nos. 02, 04, 14, and 18 received only treatment A and did not receive treatments B and C
- Subject no. 03 received only treatments A and D and did not receive treatments B and C
- Subject no. 12 received only treatment A, B, and D and did not receive treatment C
- Subject no. 22 received only treatment C and subject nos. 23 and 24 received only treatment C and D. All these subjects did not receive treatments A and B

**Fig. 2:**
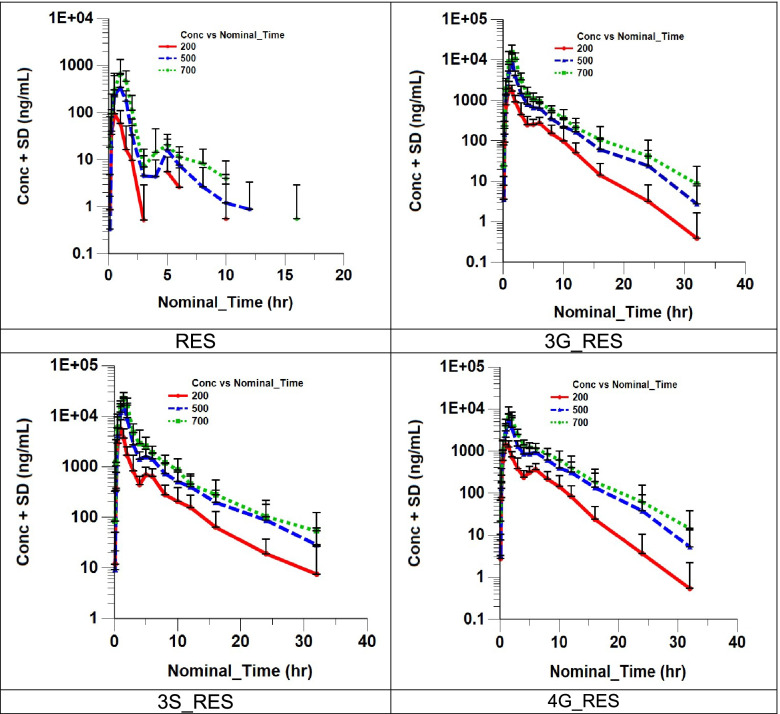
Log-linear concentration (ng/mL) vs. time plots of Resveratrol (RES) and its metabolites Resveratrol Sulfate (3S_RES), Resveratrol 3-Glucuronide (3G_RES) and Resveratrol 4-Glucuronide (4G_RES) in Human Plasma

**Fig. 3:**
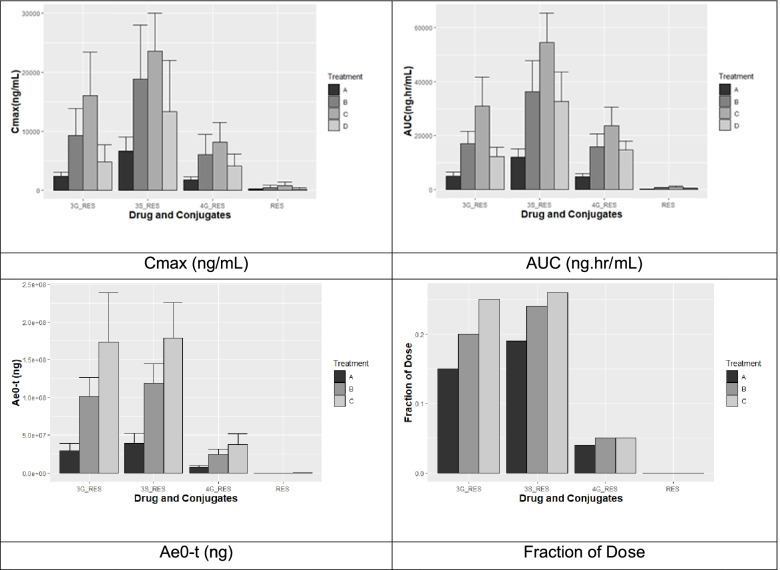
Column plots of Cmax (ng/mL), AUC (ng.hr/mL), amount (Ae0-t (ng) ) and fraction collected in urine of RES, 3_RES, 3S_RES, and 4G_RES

Plasma concentrations are summarized in Fig. 2. PK parameters are summarized in Table 2 and compared in Fig. 3. Plasma concentration data, urine concentration data, and descriptive statistics of plasma and urine pharmacokinetic parameters by treatment for resveratrol and metabolites in the PK population are available upon request.

**Table 2:** Summary of plasma pharmacokinetic parameters of JOTROL^TM^ (resveratrol) (PK population)

		Treatment A				Treatment B			
Analyte	Parameter (unit)	*N*	Mean	SD	CV%	*N*	Mean	SD	CV%
Resveratrol	AUC_0-t_ (h*ng/mL)	21	149	108	72	16	480	256	53
Plasma	AUC_0-inf_ (h*ng/mL)	1	215	-	-	5	574	325	57
	Residual Area (%)	1	5.48	-	-	5	5.55	4.08	74
	C_max_ (ng/mL)	21	127	116	91	16	455	409	90
	T_1/2 el_ (h)	1	1.26	-	-	5	2.74	1.71	63
	K_el_ (/h)	1	0.552	-	-	5	0.332	0.162	49
		Treatment A				Treatment B			
Analyte	Parameter (unit)	*N*	Median	Min	Max	*N*	Median	Min	Max
Resveratrol	T_max_ (h)	21	0.999	0.25	2.01	16	1	0.496	2.001
		Treatment A				Treatment B			
Analyte	Parameter (unit)	*N*	Mean	SD	CV%	*N*	Mean	SD	CV%
Urine	Ae0-t (ng)	21	22700	17100	75	16	54300	27100	50
	Rmax (ng/h)	21	12100	10000	82	16	39300	53700	137
	TRmax (h)	21	1.5	2.49	166	16	0.678	0.384	57
	CLR (L/h)	21	0.25	0.28	112	16	0.14	0.09	63
		Treatment C				Treatment D			
Analyte	Parameter (unit)	*N*	Mean	SD	CV%	*N*	Mean	SD	CV%
Resveratrol	AUC_0-t_ (h*ng/mL)	18	886	456	52	14	306	201	66
Plasma	AUC_0-inf_ (h*ng/mL)	3	611	172	28	2	613	373	61
	Residual Area (%)	3	3.76	1.45	39	2	3.7	4.12	111
	C_max_ (ng/mL)	18	805	590	73	14	205	207	101
	T_1/2 el_ (h)	3	1.6	0.4	25	2	1.71	1.22	72
	K_el_ (/h)	3	0.455	0.13	29	2	0.547	0.392	72
		Treatment C				Treatment D			
Analyte	Parameter (unit)	*N*	Median	Min	Max	*N*	Median	Min	Max
Resveratrol	T_max_ (h)	18	1.025	0.251	2	14	1.497	0.499	2.002
		Treatment C							
Analyte	Parameter (unit)	*N*	Mean	SD	CV%				
Urine	Ae0-t (ng)	18	143000	127000	88				
	Rmax (ng/h)	18	63400	57700	91				
	TRmax (h)	18	0.974	0.796	82				
	CLR (L/h)	18	0.19	0.18	95				

No urinary data collected in treatment D

*N* number of observations, *SD* standard deviation, *CV* coefficient of variation, *Min* minimum, *Max* maximum

The absorption of resveratrol was similar for all tested doses after a fast, with Tmax values ~ 1 h after dosing. After a meal, the Tmax increased to ~ 1.5 h. Mean AUC_0-t_ increased with increasing resveratrol doses, with mean values ranging from 149 to 886 h*ng/mL, while the mean Cmax ranged from 127 to 805 ng/mL. Using a power model to test for dose linearity, the increase in exposure was higher than expected if dose proportional. There are gender differences in the exposure of resveratrol (*p* = 0.011). The mean female AUC values tend to be higher than male values at all doses. After a 500 mg dose and high fat meal, the mean AUC_0-t_ decreased from the value for a 500 mg dose after a fast, with mean value of 306 (vs 480) h*ng/mL and the mean Cmax of 205 (vs 455) ng/mL. This study demonstrates that the consumption of food prior to dosing affects the PK of resveratrol by lowering the rate and extent of absorption as the 90% geometric CI obtained for AUC_0-t_ and Cmax were not within the acceptance range and delayed the peak concentration by approximately 30 min. Relative to the three conjugates, resveratrol plasma concentrations were very low and the drug was eliminated rapidly, with a T½ of ~ 2 h. The T½ values (as well as parameters also derived from the elimination rate constant) listed above should be interpreted with caution as N was low for most of the groups when calculating AUC0-inf, T1/2, and Kel. This was due (mostly) to later timepoints falling below the lower limit of quantification. Renal clearance was low, ranging from 0.14 to 0.25 L/h (~ 4 mL/min) and was similar between all treatments indicating a non-renal elimination pathway.

Details of the conjugate PK parameters are found in data on file. The median Tmax of resveratrol-3-glucuronide was similar for all tested doses ranging from 1.006 to 1.505 h post-dose. The mean resveratrol-3-glucuronide AUC_0-t_ increased with increase in resveratrol doses with mean values ranging from 4970 to 31000 h*ng/mL and while the mean Cmax ranged from 2390 to 16000 ng/mL. The median Tmax of resveratrol-4′-glucuronide was similar for all tested doses, ranging from 1.01 to 1.51 h post-dose. The mean resveratrol-4′-glucuronide AUC_0-t_ increased with increase in resveratrol doses, with mean values ranging from 4700 to 23600 h*ng/mL, and the mean Cmax ranged from 1710 to 8190 ng/mL The median Tmax of resveratrol-3-sulfate was similar for all tested doses ranging from 1.00 to 1.50 h post-dose. The mean resveratrol-3-sulfate AUC_0-t_ increased with increasing resveratrol doses, with mean values ranging from 12000 to 54500 h*ng/mL, while the mean Cmax ranged from 6620 to 23600 ng/mL. These three metabolites account for 40 to 55% of the total dose. Dose proportionality could not be concluded for any of these metabolites. Food consumption significantly decreased the concentrations for resveratrol-3-sulfate and resveratrol-3-glucuronide but not resveratrol-4′-glucuronide.

### Safety and tolerability

A summary of exposure by treatment is presented below in Table 3.

**Table 3:** Safety population and extent of exposure

	Part 1			Part 2	Overall
	Treatment A	Treatment B	Treatment C	Treatment D	
Number of subjects who received at least one dose, *N*	21	16	18	15	24
Female	14 (66.7)	10 (62.5)	10 (55.6)	8 (53.3)	14 (58.3)
Male	7 (33.3)	6 (37.5)	8 (44.4)	7 (46.7)	10 (41.7)
Number of subjects who completed each treatment period, *n* (%)	16 (76.2%)	15 (93.8%)	15 (83.3%)	14 (93.3%)	–
Number of subjects who completed all treatment periods (ABCD), *n* (%)	–	–	–	–	11 (45.8%)
Number of subjects who completed all part 1 treatment periods (ABC), *n* (%)	–	–	–	–	15 (62.5%)
Number of subjects who completed at least one treatment period, *n* (%)	–	–	–	–	24 (100%)

*N* number of subjects dosed, *n (%)* number of subjects included compared to the number of subjects dosed

### Adverse events

#### Brief summary of adverse events

A total of 25 treatment emergent adverse events (TEAEs) were reported by 15 (62.5%) of the 24 subjects who received any amount of study drug. Nine (9) TEAEs were reported by 7 (33.3%) of the 21 subjects who received treatment A, 4 TEAEs were reported by 4 (25.0%) of the 16 subjects who received treatment B, 6 TEAEs were reported by 6 (33.3%) of the 18 subjects who received treatment C, and 6 TEAEs were reported by 6 (40.0%) of the 15 subjects who received treatment D.

The highest frequency of subjects experiencing TEAEs was observed in the treatment D (40.0%) followed by treatment A and treatment C (33.3%, each) and treatment B (25.0%). The number of subjects who reported TEAEs was not notably different between the treatments A, B, and C. No clear trend was observed with number of TEAEs reported with increasing doses of resveratrol. Although the number of TEAEs were not notably different between treatment B (fasting) and treatment D (fed), there was a slight difference in the proportion of subjects who reported TEAEs.

Around half (13/25; 52%) of all TEAEs were related to the study drug. There were no deaths, serious, or severe TEAEs reported. Most (23) TEAEs reported were mild in severity. Two (2) subjects reported moderate TEAEs: 1 subject after receiving treatment A (COVID-19) and 1 subject after receiving treatment C (syncope). Three (3) subjects were temporarily or permanently withdrawn from the study due to TEAEs: subject no. 07 was withdrawn due to TEAE of SARS-CoV-2 test positive, and the study drug was interrupted for 2 subjects due to TEAEs; subject nos. 02 and 03 due to TEAE of COVID-19. One (1) subject (subject no. 04) was withdrawn due to the non-TEAE event back pain, which was pre-existing. Subject no. 15 experienced vomiting and was withdrawn from the study; however, this was due to the potential impact on PK analysis, rather than as a direct result of the TEAE. Majority of the TEAEs were resolved by the end of the study. A summary of TEAE frequencies by cohort is presented in Table 4.

**Table 4:** Summary of adverse events

	Part 1			Part 2	Overall
	Treatment A	Treatment B	Treatment C	Treatment D	
Number of subjects dosed, *N*	21	16	18	15	24
Number of subjects with at least one TEAE, *N* (%)	7 (33.3%)	4 (25.0%)	6 (33.3%)	6 (40.0%)	15 (62.5%)
Number of TEAEs, *n*	9	4	6	6	25
Number of serious TEAEs, *n*	0	0	0	0	0
Number of severe TEAEs, *n*	0	0	0	0	0
Number of related TEAEs^a^, *n*	6	3	1	3	13
Number of subjects who discontinued due to TEAEs, *n* (%)	3 (60.0%)	0	1 (33.3%)	0	4 (40.0%)
Number of subjects who discontinued permanently due to non-TEAEs, *n* (%)	1 (11.1%)	0	0	0	1 (4.2%)
Number of deaths, *n*	0	0	0	0	0

^a^Includes possibly related to study medication. There were no (probably) related TEAEs

*N* number of subjects dosed, *n (%)* number and percent of subjects with TEAEs, *TEAEs* treatment-emergent adverse events

There have been over 50 publications related to “toxicity of resveratrol conjugates” since 2002 found in PubMed. Resveratrol is available OTC. Generally, no significant adverse events were observed with doses up to 5 g, so safety of conjugates does not appear to be an issue.

#### Display of adverse events

The TEAEs presented in Table 5 below were observed during this study by more than one subject who received the study medication in at least one treatment group.

**Table 5:** Most frequently reported treatment-emergent adverse events

System organ class (SOC)	Stat.	Treatment				
Preferred term		A	B	C	D	Overall
Number of subjects dosed	*N*	21	16	18	15	24
Number of TEAEs	E	21	16	18	15	24
Number of subjects with TEAEs	*n* (%)	7 (33.3)	4 (25.0)	6 (33.3)	6 (40.0)	15 (62.5)
Nervous system disorders	*n* (%) E	5 (23.8) 6	4 (25.0) 4	3 (16.7) 3	2 (13.3) 2	11 (45.8) 15
Somnolence	*n* (%) E	3 (14.3) 3	2 (12.5) 2	2 (11.1) 2	2 (13.3) 2	7 (29.2) 9
Headache	*n* (%) E	3 (14.3) 3	2 (12.5) 2	0	0	5 (20.8) 5
Infections and infestations	*n* (%) E	2 (9.5) 2	0	0	0	2 (8.3) 2
COVID-19	*n* (%) E	2 (9.5) 2	0	0	0	2 (8.3) 2

The AEs included in this table were observed by more than one subject who received the study medication

Each subject could only contribute once to each of the incidence rates, regardless of the number of occurrences

Overall: Included results from all treatment groups

*E* number of TEAEs, *N* number of subjects dosed, *n (%)* number and percent of subjects with TEAE

The most commonly reported TEAEs during this study were in the SOC of nervous system disorders. TEAEs reported in more than one subject in at least one treatment group were the following:

- Somnolence, reported by 7 (29.2%) subjects overall: by 3 (14.3%) subjects after receiving treatment A, 2 (13.3%) subjects after receiving treatment D, 2 (12.5%) subjects after receiving treatment B, and 2 (11.1%) subjects after receiving treatment C. Subject no. 13 experienced somnolence after receiving all treatments (A, B, C, and D). Other subjects experienced this event on only one occasion.
- Headache, reported by 5 (20.8%) subjects overall: by 3 (14.3%) subjects after receiving treatment A and 2 (12.5%) subjects after receiving treatment B.
- COVID-19, reported by 2 (8.3%) subjects overall: by 2 (9.5%) subjects after receiving treatment A. In addition, 1 subject reported TEAE SARS-CoV-2 test positive, under SOC Investigations.

All other TEAEs were each reported by no more than 1 subject by treatment group. Summaries of TEAEs by MedDRA® PT, SOC, treatment group, maximal severity, resolution, and relationship are available upon request. Each subject could only contribute once to each of the incidence rates. In the event of multiple occurrences for a subject, the maximal severity/relationship was used in the determination of the incidence rates.

#### Analysis of adverse events

Inferential statistical analysis of TEAEs was neither planned nor performed.

The severity of TEAEs was graded according to the following categories: mild, moderate, or severe. Of the 25 TEAEs reported, 23 were graded as mild, and 2 were graded as moderate. None were graded as severe. Moderate TEAEs were reported by 1 subject (subject no. 02) after receiving treatment A (COVID-19) and 1 subject (subject no. 15) after receiving treatment C (syncope). Both these moderate TEAEs were recovered/resolved by the end of the study. A summary of TEAEs by severity and treatment is presented in Table 6.

**Table 6:** Frequency of treatment-emergent adverse events by severity

Treatment	Severity		
	Mild	Moderate	Severe
Treatment A	8	1	0
Treatment B	4	0	0
Treatment C	5	1	0
Treatment D	6	0	0
Overall	23	2	0

The PI or a medical sub-investigator judged the relationship of each TEAE to the study medication using the following categories: unrelated (not related), possible, probable, and remote. Overall, of the 25 TEAEs reported, the relationship of 13 TEAEs was judged as possibly related, 8 as remotely related, and 4 as unrelated. Somnolence (6 [25%] subjects; 8 events) and headache (4 [16.7%] subjects; 4 events) were the most frequent events judged to be possibly related. A summary of TEAEs by relationship and treatment is presented in Table 7.

**Table 7:** Frequency of treatment-emergent adverse events by relationship

Treatment	Relationship			
	Probably related	Possibly related	Remotely related	Unrelated
Treatment A	0	6	1	2
Treatment B	0	3	1	0
Treatment C	0	1	3	2
Treatment D	0	3	3	0
Overall	0	13	8	4

#### Deaths, other serious adverse events, and other significant adverse events

No deaths were reported during the study. There were no SAEs reported during the study. There were 3 significant AEs that led to study drug interruption/withdrawal from the study. Subject nos. 02 and 03 experienced COVID-19 after completion of period 1 and subject no. 07 reported SARS-CoV-2 test positive in period 3.

There were no clinically significant changes in ECG and no TEAEs related to ECG were reported during the study. There have been over 50 publications related to “toxicity of resveratrol conjugates” since 2002 found in PubMed. Resveratrol is available OTC. Generally, no significant adverse events were observed with doses up to 5 g, so safety of conjugates does not appear to be an issue.

## Discussion

Based on the power model analysis, the resveratrol exposures (AUC and Cmax) increased with increasing dose. However, this increase appeared to be higher than dose-proportional for AUC_0-t_ and Cmax. Comparing the Cmax in this study at 500 mg with published values in Table 3 below, there is a 1.4- to 21-fold increase in the Cmax with JOTROL^TM^, with a concomitant decrease in the amount of dose-related material in the gut. What is also seen is a high amount of variability among different dose forms and within and between studies. A standardized highly bioavailable and well-tolerated dose form is needed for further clinical development. Based on the results of this study, the product development of JOTROL^TM^ is a major step in that direction (Table 8).

**Table 8:** A summary of published clinical pharmacokinetics compared to the PK derived from the data with this study

Paper	Dose form	Mean ± SD (or range where indicated)				Comment
		Cmax	Tmax	AUC (0-24)	t1/2	
		(ng/mL)	(h)	(ng*h/mL)	(h)	
Howells et al. (2011)	5 g, SRT501, < 5 μm	1942 ± 1422	2.8 ± 1.1	6327 ± 2247	1.1 ± 0.4	
la Porte et al. (2010)	500 mg extract Transmax capsule, 2 g, standard breakfast	1274 ± 790	3.0 to 4.5	3558 ± 2195	2.4 ± 1.4	Low fat fed
la Porte et al. (2010)	+ breakfast, AUC to 12 h	689 ± 345	4.5 to 5.0	1966 ± 643	2.5 ± 0.8	High fat fed
Brown et al. (2010)	500 mg caplets, 5 g	967 ± 517	0.5 to 1.5	4097 ± 4384	7.85 ± 1.97	
Boocock et al. (2007)	500 mg caplets, 5 g, AUC to infinity	538 ± 391	0.67 to 5.0	1319 ± 780	8.5 ± 8.2	
Pollack et al. (2017)	1 g RevGenetics capsules (< 2.5 μm)	~ 400 ± 1800	3	nr	nr	Mixed meal
Yiu et al. (2015)	500 mg capsules, Mega Resveratrol, 2.5 g	261 ± 242	1.5	nr	nr	Low fat fed
Turner et al. (2015)	2 g, 500 mg encapsulated	85 ± 184	1.5	nr	15	
Kemper (this manuscript)	100 mg gelcaps, 200 mg JOTROL^TM^	127 ± 116	1.0	149 ± 108	1.26	Fasted
	100 mg gelcaps, 500 mg JOTROL^TM^	455 ± 409	1.0	480 ± 256	2.7	Fasted
	100 mg gelcaps, 700 mg JOTROL^TM^	805 ± 590	1.02	886 ± 456	1.6	Fasted
	100 mg gelcaps, 500 mg JOTROL^TM^	205 ± 207	1.7	306 ± 201	1.7	Fed
Walle et al. (2004)	25 mg (overnight fast +3 h)	Cmax <5 ng/mL; total plasma radioactivity 491 ng/mL				
	Radiolabeled dose	Oral		IV		
		Urine	Feces	Urine	Feces	
		70.5 ± 4.3	12.7 ± 6.1	64.1 ± 7.7	10.4 ± 3.7	

As seen in the Brown and LaPorte studies, a meal can retard and decrease the amount of bioavailable drug and the peak exposure appeared to be delayed by food approximately 30 min. Fasting is therefore called for before dosing. This may be due to a decrease in absorption, as the AUC and Cmax of the conjugates, which account for 40 to 55% of the dose in urine (out of a total of 70.5%, Walle above), are lower after a meal than after fasting. The amount recovered in urine is higher with increasing doses. In this study, drug and major metabolite amount in urine accounted for 38% at a 200 mg but increases to 56% at 700 mg. The amount of drug-related material in the urine is reflective of the absorption. Given that JOTROL^TM^ is a micellar formulation that may bypass the first-pass effect, allowing access to lymphatic distribution, the addition of food as a competitive sink for resveratrol was expected.

Care must be taken in the handling of plasma and urine samples. While phenolic glucuronides and sulfates are generally stable and do not deconjugate to the aglycone, only a 1% breakdown to the aglycone of a conjugate could have a significant effect on concentrations of resveratrol. As resveratrol has a small renal clearance (3% of GFR), metabolism is the major elimination path. Interruption of this path by a glucuronide formation inhibitor (such as the UGT inhibitor probenecid) could have major consequences.

## Conclusion

As the target level mean Cmax of 300 ng/ml (or more) in blood plasma resveratrol was achieved without approaching an upper limit AUC of 2100 ng*h/ml, the objective of identifying an obtainable and suitable/well-tolerated dose of JOTROL^TM^ to guide therapeutic investigations was achieved. This exposure level of resveratrol is associated the presence of elevated levels of iduronidase in fibroblasts of MPSI patients and in blood specimens of normal subjects exposed to oral resveratrol (Brothers SB et al., personal communication). Recent reports support the investigation of resveratrol in MPSI^8^; Jupiter intends to pursue these observations and to include population PK investigations in further studies to explore the PK/PD relationships in our indications of interest.

## Supplementary Information

**Additional file 1.**

## Data Availability

The datasets used and/or analyzed during the current study are available from the corresponding author on reasonable request.

## Abbreviations

AE: Adverse event
Ae0-t: Urinary excretion from time 0 to time t
ANOVA: Analysis of variance
API: Active Pharmaceutical Ingredient
AUC: Area under the time concentration profile for a given subject and dose
AUC_0-t_: AUC from time 0 to the time of the last measurable concentration
AUC0-inf: AUC0-t + Clast/Kel
BLQ: Below limit of quantitation
CAS: Chemical Abstracts Service
CI: Confidence level
CLR: Renal clearance
Cmax: Maximum concentration observed for a given subject profile and dose
cP: Centipoise, a unit of measurement for viscosity equivalent to one-hundredth of a poise
DSMB: Data and Safety Monitoring Board DSMB
E: Number of TEASs
ECG: Electro cardiogram
FIH: First in human study
Kel: Elimination rate constant
MELAS: Mitochondrial Encephalopathy, Lactic Acidosis, and Stroke-like episodes
MCT: Medium chain triglyceride
MPS 1: Mucopolysaccharidosis type 1
*N* (*n*): Number
NCA: Noncompartmental analysis
NRV: No reportable value
OTC: Over the counter
PK: Pharmacokinetics (PK)
Rmax: Maximum rate of urinary elimination
SAD: Single ascending doses
SRC: Scientific Review Committee
T1/2 el: Elimination half-life
TEAE(s): Treatment emergent adverse events
Tmax: Time the Cmax is observed
Trmax: Renal Tmax
