# Supplementary material for: Safety and pharmacokinetics of a highly bioavailable resveratrol preparation (JOTROL TM)
Source: AAPS Open. 2022 Jun 30;8(1):11. doi: 10.1186/s41120-022-00058-1 (PMC9243782; doi:10.1186/s41120-022-00058-1)
Supplement: Supplementary file 1 — Additional file 1. [file 41120_2022_58_MOESM1_ESM.pdf]

Table 5. Measured Values of Resveratrol, Resveratrol Sulfate, Resveratrol 3-Glucuronide and Resveratrol 4-Glucuronide in Human Urine Samples

Period	Subject	Day	Timepoint	Nominal Time	Resveratrol Result (ng/mL)	Resveratrol Sulfate Result (ng/mL)	Resveratrol 3-Glucuronide Result (ng/mL)	Resveratrol 4-Glucuronide Result (ng/mL)
1	001	1	Predose	0	BQ	BQ	BQ	BQ
1	001	1	0H-4H	4	25.9	79000	77700	7170
1	001	1	4H-8H	8	BQ	6820	8720	2210
1	001	1	8H-12H	12	BQ	826	1080	453
1	001	1	12H-24H	24	BQ	993	804	399
1	001	1	24H-32H	32	BQ	137	41.8	22.9
2	001	1	Predose	0	BQ	BQ	BQ	BQ
2	001	1	0H-4H	4	64.7	162000	148000	26100
2	001	1	4H-8H	8	11.8	38400	41200	12400
2	001	1	8H-12H	12	BQ	10100	13200	7520
2	001	1	12H-24H	24	5.07	5130	4630	2590
2	001	1	24H-32H	32	BQ	1030	646	314
3	001	1	Predose	0	BQ	BQ	BQ	BQ
3	001	1	0H-4H	4	206	547000	540000	86300
3	001	1	4H-8H	8	9.10	33700	40100	10700
3	001	1	8H-12H	12	BQ	16300	18200	7770
3	001	1	12H-24H	24	14.5	6320	6730	3130
3	001	1	24H-32H	32	BQ	2030	2100	786
1	002	1	Predose	0	BQ	BQ	BQ	BQ
1	002	1	0H-4H	4	7.17	14800	8100	1720
1	002	1	4H-8H	8	15.7	16500	9860	2590
1	002	1	8H-12H	12	BQ	5290	3310	1800
1	002	1	12H-24H	24	BQ	1750	568	361
1	002	1	24H-32H	32	BQ	398	37.7	40.1

Table 5. Measured Values of Resveratrol, Resveratrol Sulfate, Resveratrol 3-Glucuronide and Resveratrol 4-Glucuronide in Human Urine Samples (Continued)

Period	Subject	Day	Timepoint	Nominal Time	Resveratrol Result (ng/mL)	Resveratrol Sulfate Result (ng/mL)	Resveratrol 3-Glucuronide Result (ng/mL)	Resveratrol 4-Glucuronide Result (ng/mL)
1	003	1	Predose	0	BQ	BQ	BQ	BQ
1	003	1	0H-4H	4	17.9	52200	18100	2870
1	003	1	4H-8H	8	5.64	13100	7290	2270
1	003	1	8H-12H	12	BQ	1610	682	265
1	003	1	12H-24H	24	BQ	658	127	50.1
1	003	1	24H-32H	32	BQ	518	75.0	BQ
1	004	1	Predose	0	BQ	BQ	BQ	BQ
1	004	1	0H-4H	4	63.6	92100	59100	11600
1	004	1	4H-8H	8	14.8	9640	9140	2920
1	004	1	8H-12H	12	BQ	3640	3490	1440
1	004	1	12H-24H	24	BQ	1300	816	435
1	004	1	24H-32H	32	BQ	703	268	130
1	005	1	Predose	0	BQ	BQ	BQ	BQ
1	005	1	0H-4H	4	16.8	42600	24800	4440
1	005	1	4H-8H	8	BQ	12400	7350	2240
1	005	1	8H-12H	12	BQ	7340	4920	1770
1	005	1	12H-24H	24	BQ	1940	976	370
1	005	1	24H-32H	32	BQ	775	137	53.1
2	005	1	Predose	0	BQ	BQ	BQ	BQ
2	005	1	0H-4H	4	64.8	165000	108000	13700
2	005	1	4H-8H	8	42.4	97900	60300	11600
2	005	1	8H-12H	12	27.6	35500	14900	6950
2	005	1	12H-24H	24	5.35	6450	3120	1240
2	005	1	24H-32H	32	BQ	1130	346	128
3	005	1	Predose	0	BQ	BQ	BQ	BQ

Table 5. Measured Values of Resveratrol, Resveratrol Sulfate, Resveratrol 3-Glucuronide and Resveratrol 4-Glucuronide in Human Urine Samples (Continued)

Period	Subject	Day	Timepoint	Nominal Time	Resveratrol Result (ng/mL)	Resveratrol Sulfate Result (ng/mL)	Resveratrol 3-Glucuronide Result (ng/mL)	Resveratrol 4-Glucuronide Result (ng/mL)
3	005	1	0H-4H	4	132	312000	211000	24600
3	005	1	4H-8H	8	132	308000	173000	28200
3	005	1	8H-12H	12	22.3	57800	34100	10700
3	005	1	12H-24H	24	18.0	13400	7380	3040
3	005	1	24H-32H	32	BQ	1670	521	212
1	006	1	Predose	0	BQ	BQ	BQ	BQ
1	006	1	0H-4H	4	8.07	50400	41100	8290
1	006	1	4H-8H	8	BQ	2850	2140	709
1	006	1	8H-12H	12	BQ	9060	4070	1660
1	006	1	12H-24H	24	BQ	1750	512	330
1	006	1	24H-32H	32	BQ	433	53.8	30.7
2	006	1	Predose	0	BQ	BQ	BQ	BQ
2	006	1	0H-4H	4	49.2	129000	131000	19600
2	006	1	4H-8H	8	7.62	29700	19500	6690
2	006	1	8H-12H	12	12.1	22900	17300	7080
2	006	1	12H-24H	24	BQ	5090	2790	1440
2	006	1	24H-32H	32	BQ	643	108	74.1
3	006	1	Predose	0	BQ	BQ	BQ	BQ
3	006	1	0H-4H	4	51.0	240000	258000	34400
3	006	1	4H-8H	8	40.7	118000	158000	34100
3	006	1	8H-12H	12	13.0	32200	30600	11100
3	006	1	12H-24H	24	5.66	6320	3520	2060
3	006	1	24H-32H	32	BQ	1070	254	161
1	007	1	Predose	0	BQ	BQ	BQ	BQ
1	007	1	0H-4H	4	7.80	27100	22200	4320

Table 5. Measured Values of Resveratrol, Resveratrol Sulfate, Resveratrol 3-Glucuronide and Resveratrol 4-Glucuronide in Human Urine Samples (Continued)

Period	Subject	Day	Timepoint	Nominal Time	Resveratrol Result (ng/mL)	Resveratrol Sulfate Result (ng/mL)	Resveratrol 3-Glucuronide Result (ng/mL)	Resveratrol 4-Glucuronide Result (ng/mL)
1	007	1	4H-8H	8	BQ	12300	6900	2490
1	007	1	8H-12H	12	BQ	4440	3180	1800
1	007	1	12H-24H	24	BQ	1310	497	433
1	007	1	24H-32H	32	BQ	513	78.4	33.8
2	007	1	Predose	0	BQ	BQ	BQ	BQ
2	007	1	0H-4H	4	41.1	101000	84900	14300
2	007	1	4H-8H	8	6.67	23700	15100	6300
2	007	1	8H-12H	12	BQ	11400	6190	4030
2	007	1	12H-24H	24	BQ	1160	298	331
2	007	1	24H-32H	32	BQ	291	49.9	35.6
3	007	1	Predose	0	BQ	BQ	BQ	BQ
3	007	1	0H-4H	4	83.3	154000	134000	20700
3	007	1	4H-8H	8	35.8	95300	66200	18100
3	007	1	8H-12H	12	8.87	36200	18200	10600
3	007	1	12H-24H	24	BQ	6370	2160	1880
3	007	1	24H-32H	32	BQ	1070	209	147
1	008	1	Predose	0	BQ	BQ	BQ	BQ
1	008	1	0H-4H	4	63.0	84600	69400	7140
1	008	1	4H-8H	8	6.73	9430	9650	1440
1	008	1	8H-12H	12	9.67	15700	12600	2900
1	008	1	12H-24H	24	BQ	4620	2340	679
1	008	1	24H-32H	32	BQ	995	141	71.2
2	008	1	Predose	0	BQ	BQ	BQ	BQ
2	008	1	0H-4H	4	39.9	107000	84600	11500
2	008	1	4H-8H	8	79.3	73600	53600	11000

Table 5. Measured Values of Resveratrol, Resveratrol Sulfate, Resveratrol 3-Glucuronide and Resveratrol 4-Glucuronide in Human Urine Samples (Continued)

Period	Subject	Day	Timepoint	Nominal Time	Resveratrol Result (ng/mL)	Resveratrol Sulfate Result (ng/mL)	Resveratrol 3-Glucuronide Result (ng/mL)	Resveratrol 4-Glucuronide Result (ng/mL)
2	008	1	8H-12H	12	14.8	32100	28000	6550
2	008	1	12H-24H	24	8.67	11000	6520	2000
2	008	1	24H-32H	32	BQ	3670	810	311
3	008	1	Predose	0	BQ	BQ	BQ	BQ
3	008	1	0H-4H	4	146	272000	183000	22300
3	008	1	4H-8H	8	17.3	39100	20900	5720
3	008	1	8H-12H	12	104	66900	38900	13500
3	008	1	12H-24H	24	31.4	10500	3380	1350
3	008	1	24H-32H	32	5.90	7770	1620	622
1	009	1	Predose	0	BQ	BQ	BQ	BQ
1	009	1	0H-4H	4	9.02	23300	13400	4190
1	009	1	4H-8H	8	BQ	8140	7850	4710
1	009	1	8H-12H	12	BQ	2530	3630	2720
1	009	1	12H-24H	24	BQ	826	379	402
1	009	1	24H-32H	32	BQ	479	150	128
2	009	1	Predose	0	BQ	BQ	BQ	BQ
2	009	1	0H-4H	4	19.2	91700	70200	15200
2	009	1	4H-8H	8	55.9	54600	52400	26000
2	009	1	8H-12H	12	BQ	17400	24800	13400
2	009	1	12H-24H	24	10.8	16000	17400	12100
2	009	1	24H-32H	32	BQ	2550	2460	1480
3	009	1	Predose	0	BQ	BQ	BQ	BQ
3	009	1	0H-4H	4	41.1	191000	175000	37100
3	009	1	4H-8H	8	49.3	65600	46800	31100
3	009	1	8H-12H	12	105	80100	73000	58000

Table 5. Measured Values of Resveratrol, Resveratrol Sulfate, Resveratrol 3-Glucuronide and Resveratrol 4-Glucuronide in Human Urine Samples (Continued)

Period	Subject	Day	Timepoint	Nominal Time	Resveratrol Result (ng/mL)	Resveratrol Sulfate Result (ng/mL)	Resveratrol 3-Glucuronide Result (ng/mL)	Resveratrol 4-Glucuronide Result (ng/mL)
3	009	1	12H-24H	24	14.3	8310	11300	10600
3	009	1	24H-32H	32	BQ	640	444	390
1	010	1	Predose	0	BQ	BQ	BQ	BQ
1	010	1	0H-4H	4	BQ	17900	10500	2170
1	010	1	4H-8H	8	19.6	25400	19200	5670
1	010	1	8H-12H	12	BQ	12600	10100	3600
1	010	1	12H-24H	24	BQ	2450	1290	566
1	010	1	24H-32H	32	BQ	612	122	64.2
2	010	1	Predose	0	BQ	BQ	BQ	BQ
2	010	1	0H-4H	4	67.1	140000	113000	17700
2	010	1	4H-8H	8	93.3	98000	9050	21100
2	010	1	8H-12H	12	10.7	24200	16500	7010
2	010	1	12H-24H	24	9.82	15000	7630	3270
2	010	1	24H-32H	32	BQ	3020	918	355
3	010	1	Predose	0	BQ	BQ	BQ	BQ
3	010	1	0H-4H	4	86.9	123000	111000	18300
3	010	1	4H-8H	8	42.8	76700	41400	17900
3	010	1	8H-12H	12	151	90500	58000	25500
3	010	1	12H-24H	24	BQ	5350	2220	1250
3	010	1	24H-32H	32	BQ	2520	706	331
1	011	1	Predose	0	BQ	BQ	BQ	BQ
1	011	1	0H-4H	4	BQ	21000	15300	3370
1	011	1	4H-8H	8	BQ	6420	6430	2580
1	011	1	8H-12H	12	BQ	7850	9390	4360
1	011	1	12H-24H	24	6.08	3120	2490	1360

Table 5. Measured Values of Resveratrol, Resveratrol Sulfate, Resveratrol 3-Glucuronide and Resveratrol 4-Glucuronide in Human Urine Samples (Continued)

Period	Subject	Day	Timepoint	Nominal Time	Resveratrol Result (ng/mL)	Resveratrol Sulfate Result (ng/mL)	Resveratrol 3-Glucuronide Result (ng/mL)	Resveratrol 4-Glucuronide Result (ng/mL)
1	011	1	24H-32H	32	BQ	237	88.2	42.5
2	011	1	Predose	0	BQ	BQ	BQ	BQ
2	011	1	0H-4H	4	22.7	109000	86100	15700
2	011	1	4H-8H	8	29.6	58800	38900	18800
2	011	1	8H-12H	12	5.94	20600	22500	12400
2	011	1	12H-24H	24	6.86	6580	7250	4450
2	011	1	24H-32H	32	BQ	732	245	182
3	011	1	Predose	0	BQ	BQ	BQ	BQ
3	011	1	0H-4H	4	128	253000	217000	34100
3	011	1	4H-8H	8	124	94800	99100	37100
3	011	1	8H-12H	12	151	61400	82500	47400
3	011	1	12H-24H	24	47.1	13900	17100	10300
3	011	1	24H-32H	32	12.5	3090	2750	1570
1	012	1	Predose	0	BQ	BQ	BQ	BQ
1	012	1	0H-4H	4	54.7	36800	70800	5830
1	012	1	4H-8H	8	9.21	12200	13500	4380
1	012	1	8H-12H	12	12.0	9500	11500	5090
1	012	1	12H-24H	24	BQ	2010	1920	930
1	012	1	24H-32H	32	BQ	435	149	66.0
2	012	1	Predose	0	BQ	BQ	BQ	BQ
2	012	1	0H-4H	4	87.9	98000	125000	18700
2	012	1	4H-8H	8	14.2	31700	35300	11900
2	012	1	8H-12H	12	8.26	10100	11300	5560
2	012	1	12H-24H	24	6.92	4840	4480	2120
2	012	1	24H-32H	32	BQ	1390	753	321

Table 5. Measured Values of Resveratrol, Resveratrol Sulfate, Resveratrol 3-Glucuronide and Resveratrol 4-Glucuronide in Human Urine Samples (Continued)

Period	Subject	Day	Timepoint	Nominal Time	Resveratrol Result (ng/mL)	Resveratrol Sulfate Result (ng/mL)	Resveratrol 3-Glucuronide Result (ng/mL)	Resveratrol 4-Glucuronide Result (ng/mL)
1	013	1	Predose	0	BQ	BQ	BQ	BQ
1	013	1	0H-4H	4	13.5	30800	25000	6010
1	013	1	4H-8H	8	BQ	4100	3640	1910
1	013	1	8H-12H	12	BQ	1420	1680	1120
1	013	1	12H-24H	24	BQ	396	141	131
1	013	1	24H-32H	32	BQ	297	80.6	30.1
2	013	1	Predose	0	BQ	BQ	BQ	BQ
2	013	1	0H-4H	4	10.6	64400	49300	10500
2	013	1	4H-8H	8	11.7	42000	29700	13400
2	013	1	8H-12H	12	BQ	4600	3220	2100
2	013	1	12H-24H	24	BQ	3540	1940	1270
2	013	1	24H-32H	32	BQ	649	252	135
3	013	1	Predose	0	BQ	BQ	BQ	BQ
3	013	1	0H-4H	4	21.4	108000	87400	18800
3	013	1	4H-8H	8	BQ	24600	15600	7390
3	013	1	8H-12H	12	6.29	18700	11800	6520
3	013	1	12H-24H	24	BQ	2650	1320	928
3	013	1	24H-32H	32	BQ	806	299	184
1	014	1	Predose	0	BQ	BQ	BQ	BQ
1	014	1	0H-4H	4	58.5	59800	43600	6430
1	014	1	4H-8H	8	9.67	10700	8520	3320
1	014	1	8H-12H	12	BQ	1700	1180	560
1	014	1	12H-24H	24	BQ	2570	1710	792
1	014	1	24H-32H	32	BQ	955	374	166
1	015	1	Predose	0	BQ	BQ	BQ	BQ

Table 5. Measured Values of Resveratrol, Resveratrol Sulfate, Resveratrol 3-Glucuronide and Resveratrol 4-Glucuronide in Human Urine Samples (Continued)

Period	Subject	Day	Timepoint	Nominal Time	Resveratrol Result (ng/mL)	Resveratrol Sulfate Result (ng/mL)	Resveratrol 3-Glucuronide Result (ng/mL)	Resveratrol 4-Glucuronide Result (ng/mL)
1	015	1	0H-4H	4	97.4	93000	96700	11400
1	015	1	4H-8H	8	6.11	6430	4580	1860
1	015	1	8H-12H	12	6.23	18100	14400	8380
1	015	1	12H-24H	24	6.86	8130	5250	3270
1	015	1	24H-32H	32	10.7	4920	3790	1990
2	015	1	Predose	0	BQ	BQ	BQ	BQ
2	015	1	0H-4H	4	66.5	164000	224000	32200
2	015	1	4H-8H	8	5.55	20300	12500	4110
2	015	1	8H-12H	12	18.8	75900	34400	17900
2	015	1	12H-24H	24	26.8	20900	14400	9390
2	015	1	24H-32H	32	BQ	2430	1310	784
3	015	1	Predose	0	BQ	BQ	BQ	BQ
3	015	1	0H-4H	4	204	351000	585000	76100
3	015	1	4H-8H	8	76.8	93700	100000	29500
3	015	1	8H-12H	12	102	97300	72200	38700
3	015	1	12H-24H	24	47.1	28100	21300	12400
3	015	1	24H-32H	32	14.4	7130	8050	4200
1	016	1	Predose	0	BQ	BQ	BQ	BQ
1	016	1	0H-4H	4	20.0	55200	41600	5720
1	016	1	4H-8H	8	BQ	3570	2930	1260
1	016	1	8H-12H	12	BQ	1420	782	526
1	016	1	12H-24H	24	BQ	1110	317	223
1	016	1	24H-32H	32	BQ	625	117	49.9
2	016	1	Predose	0	BQ	BQ	BQ	BQ
2	016	1	0H-4H	4	24.7	96700	117000	9910

Table 5. Measured Values of Resveratrol, Resveratrol Sulfate, Resveratrol 3-Glucuronide and Resveratrol 4-Glucuronide in Human Urine Samples (Continued)

Period	Subject	Day	Timepoint	Nominal Time	Resveratrol Result (ng/mL)	Resveratrol Sulfate Result (ng/mL)	Resveratrol 3-Glucuronide Result (ng/mL)	Resveratrol 4-Glucuronide Result (ng/mL)
2	016	1	4H-8H	8	BQ	17900	13100	4960
2	016	1	8H-12H	12	BQ	5220	3120	1650
2	016	1	12H-24H	24	BQ	2130	883	476
2	016	1	24H-32H	32	BQ	509	84.3	42.2
3	016	1	Predose	0	BQ	BQ	BQ	BQ
3	016	1	0H-4H	4	210	244000	377000	38000
3	016	1	4H-8H	8	6.23	22600	19400	6150
3	016	1	8H-12H	12	5.41	12000	8150	3590
3	016	1	12H-24H	24	BQ	2500	1180	594
3	016	1	24H-32H	32	BQ	1190	336	141
1	017	1	Predose	0	BQ	BQ	BQ	BQ
1	017	1	0H-4H	4	111	82700	80700	17500
1	017	1	4H-8H	8	12.9	12900	9200	3920
1	017	1	8H-12H	12	BQ	3270	2370	1690
1	017	1	12H-24H	24	BQ	1240	334	365
1	017	1	24H-32H	32	BQ	254	21.8	22.4
2	017	1	Predose	0	BQ	BQ	BQ	BQ
2	017	1	0H-4H	4	37.7	124000	86200	16900
2	017	1	4H-8H	8	6.80	28200	15500	8260
2	017	1	8H-12H	12	5.63	18900	9080	7730
2	017	1	12H-24H	24	24.4	8940	2830	2180
2	017	1	24H-32H	32	BQ	640	105	82.0
3	017	1	Predose	0	BQ	BQ	BQ	BQ
3	017	1	0H-4H	4	226	257000	184000	29400
3	017	1	4H-8H	8	15.6	50100	22000	12800

Table 5. Measured Values of Resveratrol, Resveratrol Sulfate, Resveratrol 3-Glucuronide and Resveratrol 4-Glucuronide in Human Urine Samples (Continued)

Period	Subject	Day	Timepoint	Nominal Time	Resveratrol Result (ng/mL)	Resveratrol Sulfate Result (ng/mL)	Resveratrol 3-Glucuronide Result (ng/mL)	Resveratrol 4-Glucuronide Result (ng/mL)
3	017	1	8H-12H	12	24.6	50700	29400	18000
3	017	1	12H-24H	24	BQ	8300	3140	2160
3	017	1	24H-32H	32	BQ	755	204	125
1	018	1	Predose	0	BQ	BQ	BQ	BQ
1	018	1	0H-4H	4	184	84600	85200	15400
1	018	1	4H-8H	8	18.0	8130	6670	3460
1	018	1	8H-12H	12	30.9	16200	15100	9430
1	018	1	12H-24H	24	8.60	2390	1080	1100
1	018	1	24H-32H	32	BQ	205	70.5	54.1
1	019	1	Predose	0	BQ	BQ	BQ	BQ
1	019	1	0H-4H	4	69.7	157000	102000	22700
1	019	1	4H-8H	8	11.4	34600	16100	8180
1	019	1	8H-12H	12	22.2	25900	17800	8810
1	019	1	12H-24H	24	11.6	7780	3420	2150
1	019	1	24H-32H	32	BQ	801	218	157
2	019	1	Predose	0	BQ	BQ	BQ	BQ
2	019	1	0H-4H	4	591	922000	1110000	172000
2	019	1	4H-8H	8	140	225000	132000	53600
2	019	1	8H-12H	12	67.3	96200	54700	24900
2	019	1	12H-24H	24	116	55000	38500	20100
2	019	1	24H-32H	32	10.3	10900	6400	3450
3	019	1	Predose	0	BQ	BQ	BQ	BQ
3	019	1	0H-4H	4	1250	469000	572000	108000
3	019	1	4H-8H	8	202	154000	99400	51200
3	019	1	8H-12H	12	72.6	81800	35300	17500

Table 5. Measured Values of Resveratrol, Resveratrol Sulfate, Resveratrol 3-Glucuronide and Resveratrol 4-Glucuronide in Human Urine Samples (Continued)

Period	Subject	Day	Timepoint	Nominal Time	Resveratrol Result (ng/mL)	Resveratrol Sulfate Result (ng/mL)	Resveratrol 3-Glucuronide Result (ng/mL)	Resveratrol 4-Glucuronide Result (ng/mL)
3	019	1	12H-24H	24	536	89000	93800	49900
3	019	1	24H-32H	32	39.2	13700	13000	7590
1	020	1	Predose	0	BQ	BQ	BQ	BQ
1	020	1	0H-4H	4	39.2	23200	28900	8860
1	020	1	4H-8H	8	141	73100	65900	35000
1	020	1	8H-12H	12	BQ	4390	3950	2780
1	020	1	12H-24H	24	BQ	1710	1400	929
1	020	1	24H-32H	32	12.1	4660	2510	1530
2	020	1	Predose	0	BQ	BQ	BQ	BQ
2	020	1	0H-4H	4	54.9	69200	72700	17400
2	020	1	4H-8H	8	62.2	58400	51900	26900
2	020	1	8H-12H	12	23.9	29800	31500	23000
2	020	1	12H-24H	24	25.0	14200	12000	9760
2	020	1	24H-32H	32	6.17	1960	1060	896
3	020	1	Predose	0	BQ	BQ	BQ	BQ
3	020	1	0H-4H	4	139	141000	177000	44300
3	020	1	4H-8H	8	15.3	31800	34600	17300
3	020	1	8H-12H	12	12.3	38500	32100	16600
3	020	1	12H-24H	24	21.9	22200	18300	9460
3	020	1	24H-32H	32	17.7	8780	6100	3410
1	021	1	Predose	0	BQ	BQ	BQ	BQ
1	021	1	0H-4H	4	30.6	64800	48800	11000
1	021	1	4H-8H	8	BQ	9550	5770	2540
1	021	1	8H-12H	12	BQ	4290	3660	1820
1	021	1	12H-24H	24	BQ	4100	2300	1210

Table 5. Measured Values of Resveratrol, Resveratrol Sulfate, Resveratrol 3-Glucuronide and Resveratrol 4-Glucuronide in Human Urine Samples (Continued)

Period	Subject	Day	Timepoint	Nominal Time	Resveratrol Result (ng/mL)	Resveratrol Sulfate Result (ng/mL)	Resveratrol 3-Glucuronide Result (ng/mL)	Resveratrol 4-Glucuronide Result (ng/mL)
1	021	1	24H-32H	32	BQ	2350	1120	542
2	021	1	Predose	0	BQ	BQ	BQ	BQ
2	021	1	0H-4H	4	58.6	208000	164000	37600
2	021	1	4H-8H	8	12.3	47100	35400	13600
2	021	1	8H-12H	12	9.79	38300	22400	15000
2	021	1	12H-24H	24	6.91	14900	9830	5520
2	021	1	24H-32H	32	BQ	9630	5000	2270
3	021	1	Predose	0	BQ	BQ	BQ	BQ
3	021	1	0H-4H	4	120	355000	359000	76900
3	021	1	4H-8H	8	18.0	57100	45300	19300
3	021	1	8H-12H	12	20.8	55500	41600	18800
3	021	1	12H-24H	24	21.1	32600	28700	13600
3	021	1	24H-32H	32	7.87	12900	10100	4080
3	022	1	Predose	0	BQ	BQ	BQ	BQ
3	022	1	0H-4H	4	77.3	189000	268000	21300
3	022	1	4H-8H	8	19.7	50900	61400	19200
3	022	1	8H-12H	12	83.8	108000	81200	29100
3	022	1	12H-24H	24	52.4	25300	21000	9250
3	022	1	24H-32H	32	BQ	3180	2080	973
3	023	1	Predose	0	BQ	BQ	BQ	BQ
3	023	1	0H-4H	4	73.2	211000	258000	34600
3	023	1	4H-8H	8	17.9	48100	45700	12400
3	023	1	8H-12H	12	32.4	26700	23700	9770
3	023	1	12H-24H	24	10.7	5710	3800	2130
3	023	1	24H-32H	32	BQ	1290	606	339

Table 5. Measured Values of Resveratrol, Resveratrol Sulfate, Resveratrol 3-Glucuronide and Resveratrol 4-Glucuronide in Human Urine Samples (Continued)

Period	Subject	Day	Timepoint	Nominal Time	Resveratrol Result (ng/mL)	Resveratrol Sulfate Result (ng/mL)	Resveratrol 3-Glucuronide Result (ng/mL)	Resveratrol 4-Glucuronide Result (ng/mL)
3	024	1	Predose	0	BQ	BQ	BQ	BQ
3	024	1	0H-4H	4	1060	543000	653000	82400
3	024	1	4H-8H	8	516	163000	156000	43600
3	024	1	8H-12H	12	378	145000	124000	50900
3	024	1	12H-24H	24	46.5	18100	11900	5420
3	024	1	24H-32H	32	BQ	3250	1340	441

BQ Value below the lower limit of quantitation (5.00 ng/mL for Resveratrol, 100 ng/mL for Resveratrol Sulfate, 20.0n ng/mL for Resveratrol 4-Glucuronide and 20.0 ng/mL for Resveratrol 4-Glucuronide)
